# Morel‐Lavallee injury a case study

**DOI:** 10.1002/ccr3.1518

**Published:** 2018-04-10

**Authors:** Karen M. Myrick, Stephen Davis

**Affiliations:** ^1^ Orthopedic Associates of Hartford Hamden Connecticut; ^2^ Quinnipiac University Hamden Connecticut

**Keywords:** Degloving, Morel‐Lavallee lesion, soft tissue injury, trauma

## Abstract

Morel‐Lavallee lesions are post‐traumatic, soft‐tissue degloving injuries commonly misdiagnosed as hematomas or ruptured bursa. The clinician needs to be aware of this injury, in order to provide appropriate patient care and treatment. If not treated early, risks include superinfection, continued expansion, overlying tissue necrosis, and suboptimal patient outcomes.

## Introduction

Degloving soft tissue injuries, the Morel‐Lavallee lesion, are a clinical problem requiring prompt and appropriate treatment. We present a case, including pathophysiology, imaging, treatment, and outcomes. Degloving soft tissue injuries are underreported and can have devastating results. Early recognition and early management are paramount to enhance patient outcomes.

The Morel‐Lavallee lesion (MLL) was initially described by a French surgeon named Maurice Morel‐Lavallee in 1853, as a closed degloving injury where the skin and superficial fascia are traumatically separated, creating a dead space.^1^, ^2^, ^3^ The mechanism of injury is the application of high‐intensity forces to the body in either a direct or tangential shearing, and the effect is a separation of the skin and subcutaneous tissue from the underlying muscle fascia.^4^, ^5^ When the traumatic injury occurs, there is damage to the vascular and lymphatic supply, which then leads to the accumulation of blood and lymph in the dead space generated by the separation of the superficial and deep fascia.^5^, ^6^, ^7^ The material inside of the lesion creates a chronic inflammatory process. These lesions are associated with significant morbidity and mortality.^4^, ^8^ Over time, there is resorption of the hemorrhagic elements, increasing the serosanguinous fluid and progressive fibrous encapsulation which hinders resorption and leads to a slow continued expansion.^6^, ^9^ The areas most likely to suffer from Morel‐Lavallee injuries are the hip/greater trochanter, anterolateral thigh, gluteal, lumbodorsal, and scapular regions.^4^, ^10^ They are usually found adjacent to osseous protuberances.^5^

In 2005, Mellado and Bencardino proposed an extensive six‐stage imaging‐based classification based on the shape of lesion, signal intensity on T1‐ and T2‐weighted images, presence of fibrous capsule, contrast enhancement, and sinus tract formation capsule^11^:

Type I Morel‐Lavallee lesion—Seroma appearing as a homogeneously hypointense on T1‐weighted image (T1WI) and hyperintense collection on T2‐weighted images (T2W2), without evidence of outer capsule formation.
Type II Morel‐Lavallee lesion—Subacute hematoma appearing as homogeneously hyperintense on both T1WI and T2WI due to the presence of methemoglobin, a characteristic of subacute hematomas.
Type III Morel‐Lavallee lesion—Chronic organizing hematomas demonstrating hypointensity on T1WI and heterogeneous hypointensity/isointensity on T2WI with capsular formation. On postcontrast sequences, Type III lesions may show capsular and internal enhancement secondary to neovascularization and granulation tissue in the organizing hematoma. This can even lead to growth over time.
Type IV Morel‐Lavallee lesion—Represents a closed laceration, with the absence of a capsule. It shows T1 hypointense and T2 hyperintense signals.
Type V Morel‐Lavallee lesion—Demonstrates a small, rounded, pseudonodular appearance and has variable T1 and T2 signal intensities.
Type VI Morel‐Lavallee lesion—Represents superimposed infection, with a thick enhancing capsule and can be associated with sinus tract.

## Case History and Examination

### History

The patient is a 50‐year‐old female who is an avid runner and martial artist. While taking her dog for a walk, the patient was pulled down three stairs, landing on her right side. The patient reported feeling pain immediately in the lateral right upper leg and some minor pain in the right elbow and knee. Ice was immediately applied to the upper lateral leg. She reports that the swelling was rapidly increasing, and approximately 5 by 7 cm in size. There was a small, superficial abrasion in the center of the noted area of swelling that required only a plastic bandage to control.

**Figure 1:**
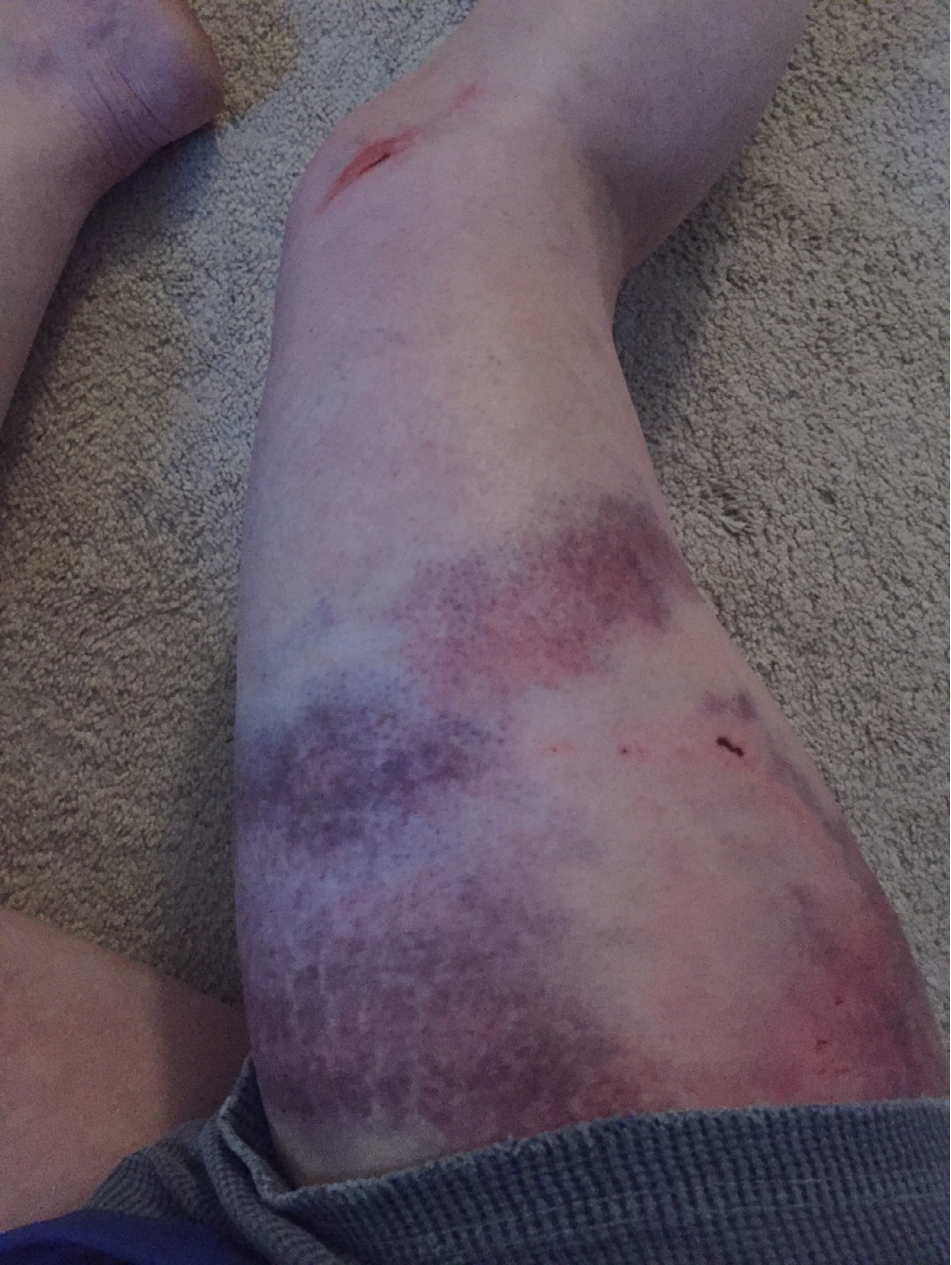
Postinjury day 1.

The following day, the area of swelling increased to approximately 6 by 8 cm, and marked ecchymosis was noted about the full length of the upper lateral leg (Fig. 1). The patient consulted with an orthopedic physician assistant, concerned with the size of the swelling. She asked the PA if he has ever seen the need for a hematoma to be drained. After reviewing the signs and symptoms and the clinical picture, the PA suggested that this might be a Morel‐Lavallee lesion, and referred the patient to the trauma service.

### Clinical examination and findings

Diagnosis of closed internal degloving injuries is based on history and physical examination. These injuries often occur in association with fractures, such as pelvic and acetabulum, femur, and tibia fractures as a result of high‐energy mechanisms. The Morel‐Lavallee lesion may also occur in isolation, as in the illustrated case. The patient complains of a focal area of swelling and may or may not have associated pain or tenderness. A sensation of a fluid collection is often described as a “water balloon” under the skin.

On examination, the lesion will often show a focal area of swelling, even in obese patients, but may be less visible in patients with a large body habitus. There may be associated ecchymosis and abrasions. In thin patients, the margins of the lesion may be readily identifiable. Significant fluctuance is a hallmark, as the lesion is filled mainly by serous fluid. Because the MLL occurs at the interface between subcutaneous fat the deep fascia, the collection will feel superficial. This is in contrast to a hematoma, which typically will be deeper, less well defined, and less fluctuant as often clotting will have occurred to a variable extent. Even so, differentiating between a MLL and a hematoma may not be possible by examination alone.

Upon presentation to the trauma surgeon on postinjury day 3, the area of swelling was recorded as 10 by 10 cm. The patient had minor discomfort and complained mostly of the large area affected, which was expanding. There was significant ecchymosis about the right upper leg and hip, and a pattern of dependent ecchymosis was noted as well. The hip and knee joints exhibited full range of motion, and muscle strength was 5 of 5 in the quadriceps and hamstrings. Hip flexors and abductors demonstrated a 4 of 5 strength test and adductors 5 of 5. The central most portion of the area of swelling demonstrated hypoesthesia.

### Differential diagnosis, investigations, and treatment

The differential diagnosis includes hematoma, ruptured bursa, and muscle tear.^12^ The differential diagnosis may present as challenging from a physical examination standpoint, as many of the presenting signs overlap. Morel‐Lavallee lesions are frequently misdiagnosed.^13^ With a thorough history, a fluid collection in a typical location and a strong clinical suspicion, the diagnosis of Morel‐Lavallee lesion is likely.^6^ Ruptured bursa would occur as a fluid collection in a known anatomic location, and the Morel‐Lavallee lesion is usually distal to joints.^5^ Ruptured bursa will typically appear as hypointense on T1W1 and hyperintense on T2W1, similar to the Type I Morel‐Lavallee.^14^ Muscle tears can be differentiated by muscle weakness on physical examination, and the prefascial location and well‐defined outline differentiate Morel‐Lavallee lesions from muscle tears and intramuscular hematomas.^5^ The distinguishing feature between a hematoma and a Morel‐Lavallee on MRI is that the Morel‐Lavallee is in the location of the interfascial plane.^14^ The physical examination will reveal more fluctuance at a deeper level than the superficial hematoma.

**Figure 2:**
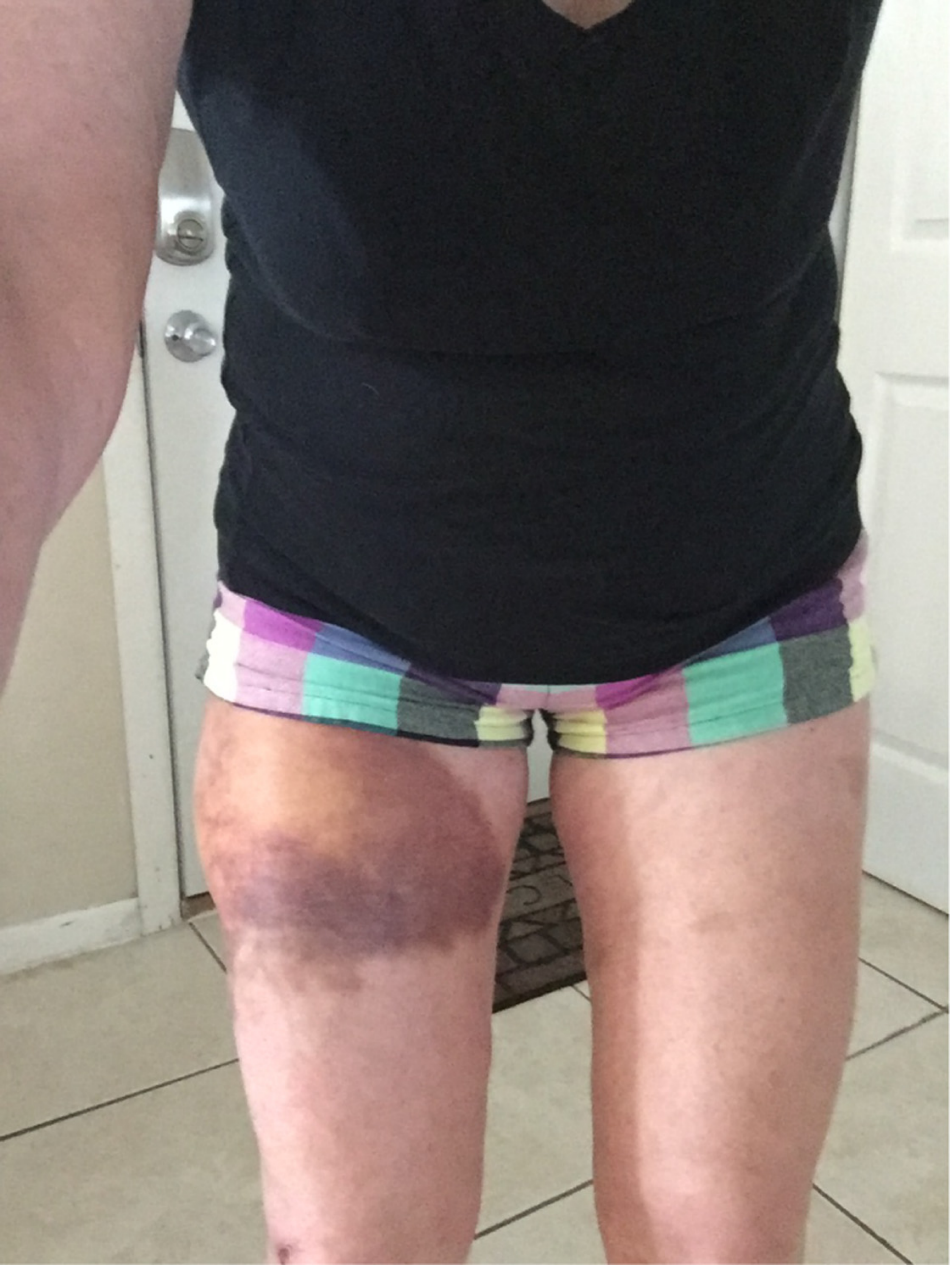
Postinjury day 6.

A period of conservative management was trialed for 17 days. In a study by Tejwani et al.,^15^ the mean time taken for Morel‐Lavallee lesions of the knee to resolve following conservative treatment was reported to be 10.6 ± 9.2 days in 27 cases. The conservative management consisted of cryotherapy with ice application five to eight times a day, 20 min on, 20 min off, compression with a 6‐inch ace bandage and elevation. Relative rest with no strenuous exercise was also undertaken. Despite the conservative measures, by day 6 postinjury, the area was noted to be slightly enlarging (Fig. 2).

**Figure 3:**
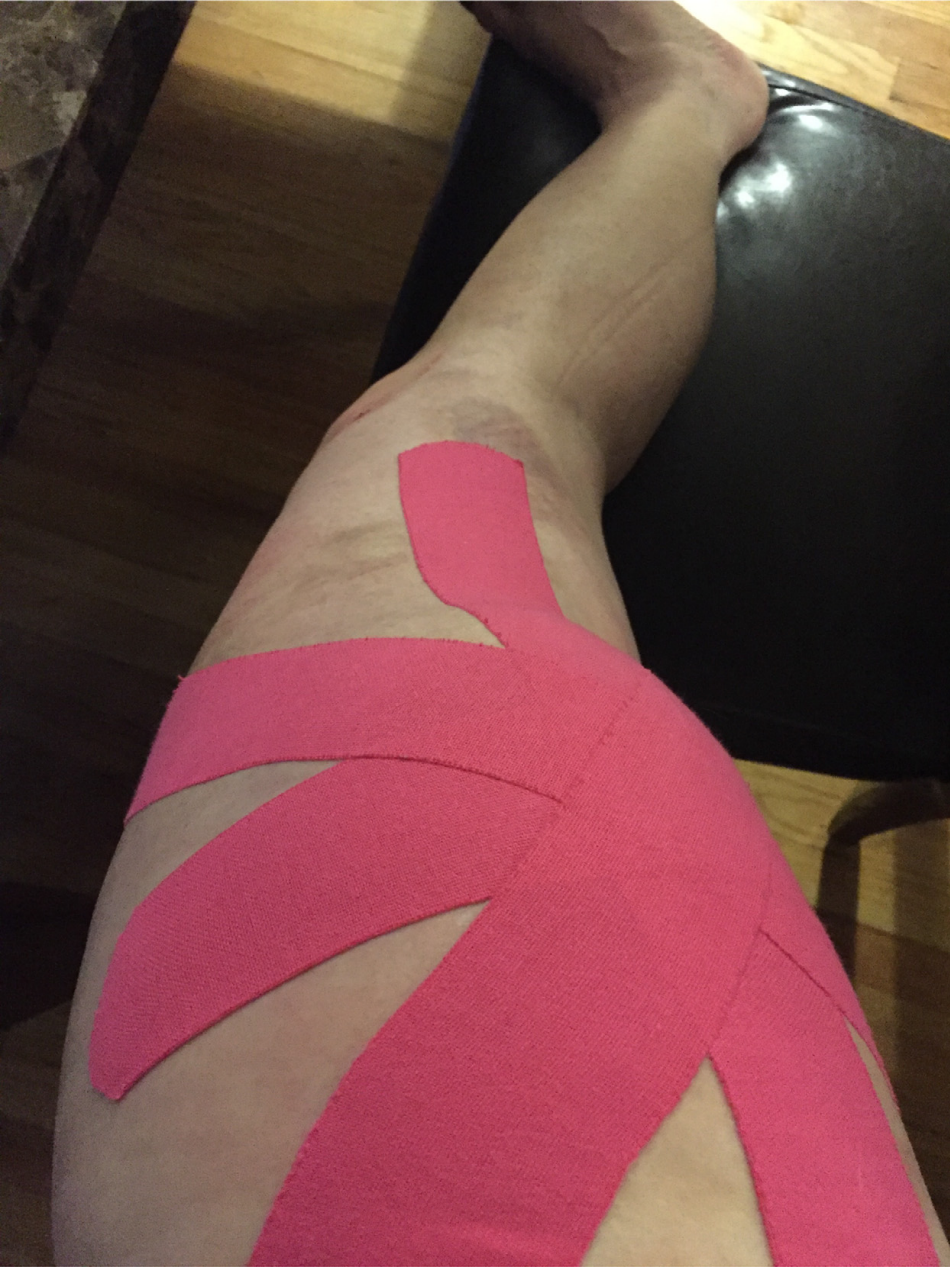
Postinjury day 14 KT tape.

By day 10 postinjury, the patient noted that there was a significant increase in the size of the area of swelling, and she called the surgeon's office. With the clinical suspicion of Morel‐Lavallee injury, an MRI was ordered and performed on postinjury day 14. After the MRI, the patient consulted with physical therapy and KT (kineseotape) was applied for comfort and in an attempt to reduce the edema (Fig. 3).

**Figure 4:**
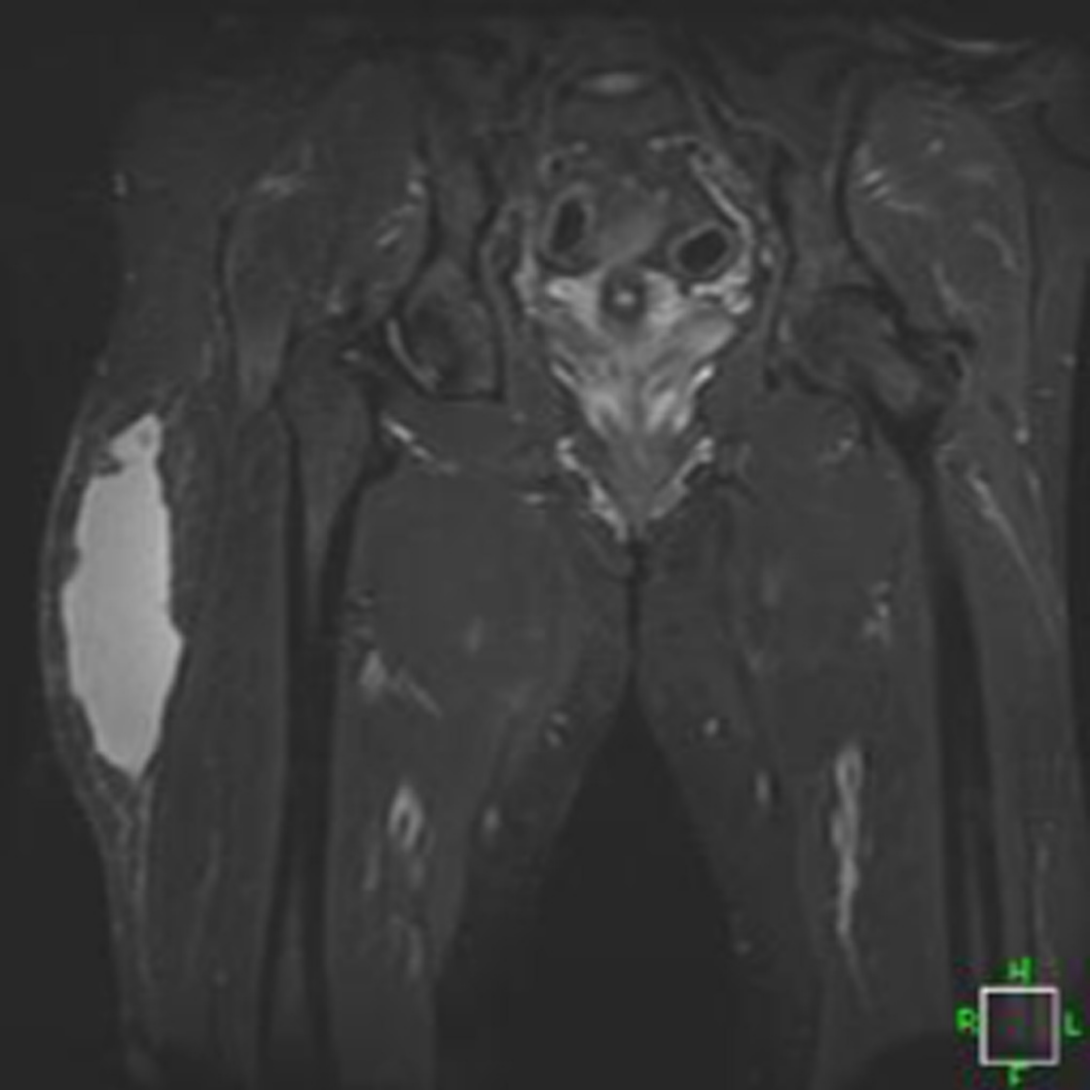
MRI.

**Figure 5:**
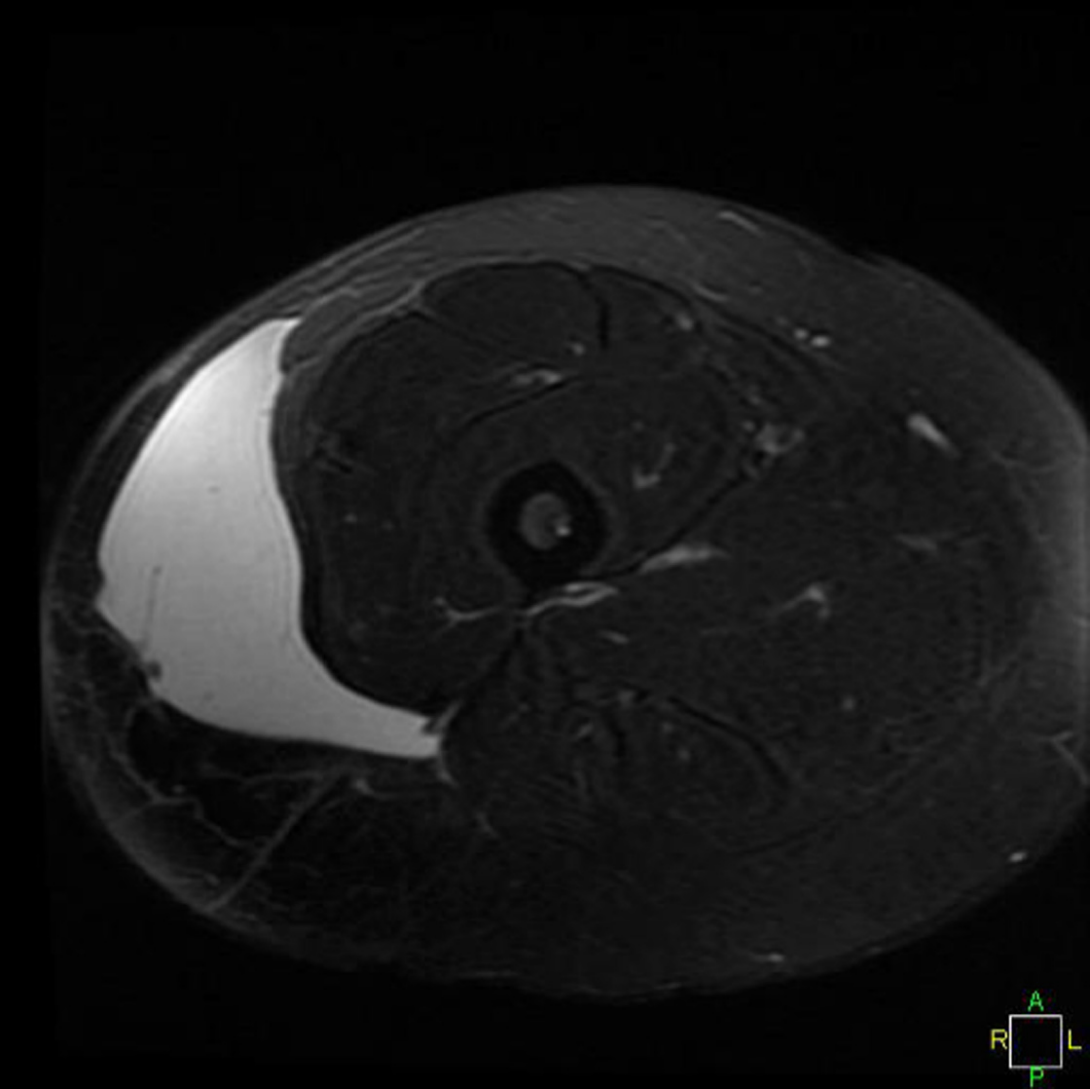
MRI.

Further evaluation of the injury can be accomplished by MRI to help confirm the diagnosis, eliminate associated injuries, and also establish the size and location of the lesion for surgical planning. Magnetic resonance imagery (MRI) was scheduled and completed on day 14. The MRI revealed a large, 15 cm by 7 cm by 5 cm area of increased intensity of T2‐weighted images. There was also a mild joint effusion and a chronically torn ligamentum teres. Musculotendinous structures were noted to be intact, and no fracture was appreciated (Figs. 4 and 5). A Type III lesion was identified.

**Figure 6:**
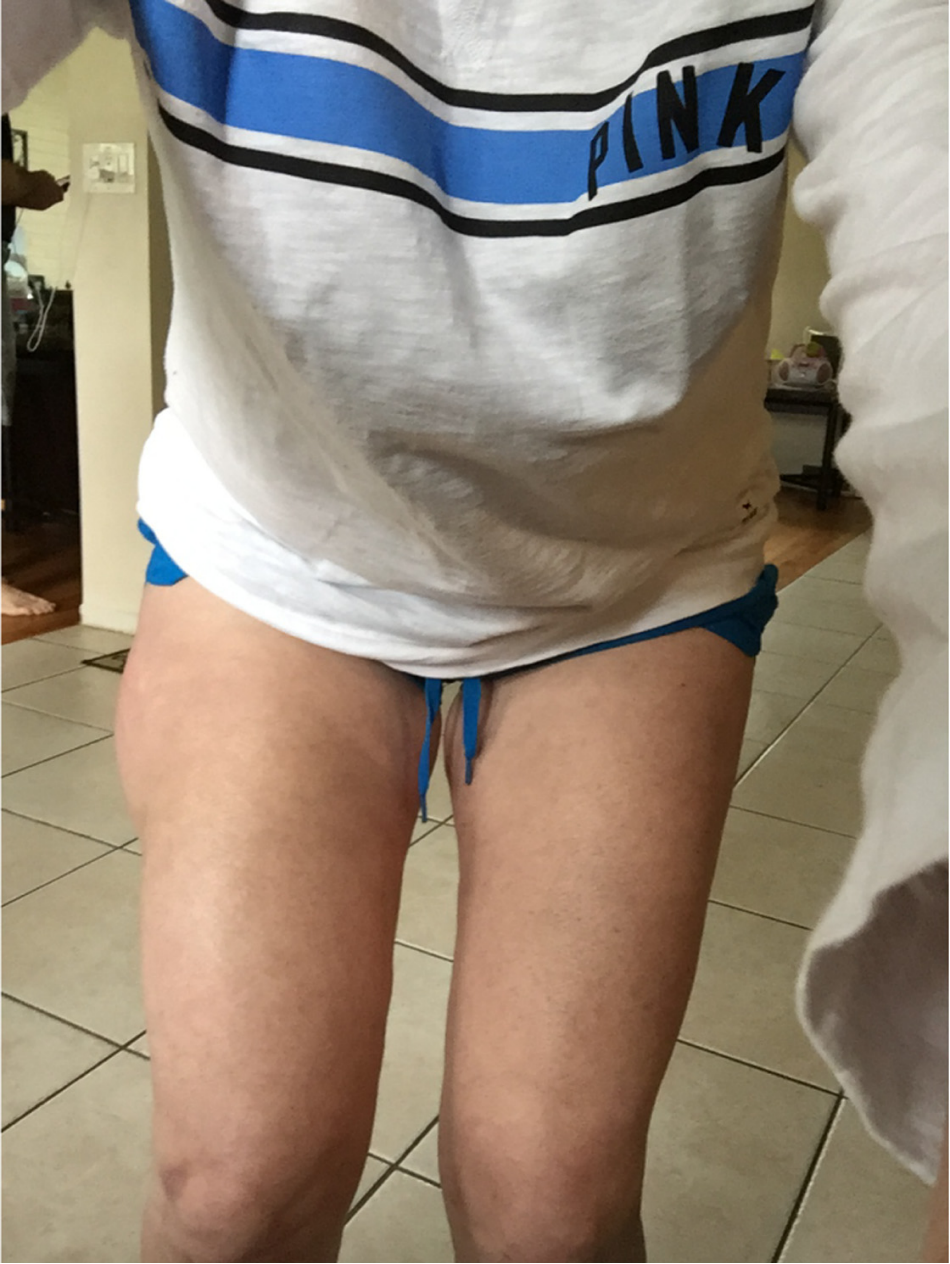
Postinjury day 23, day of surgery.

**Figure 7:**
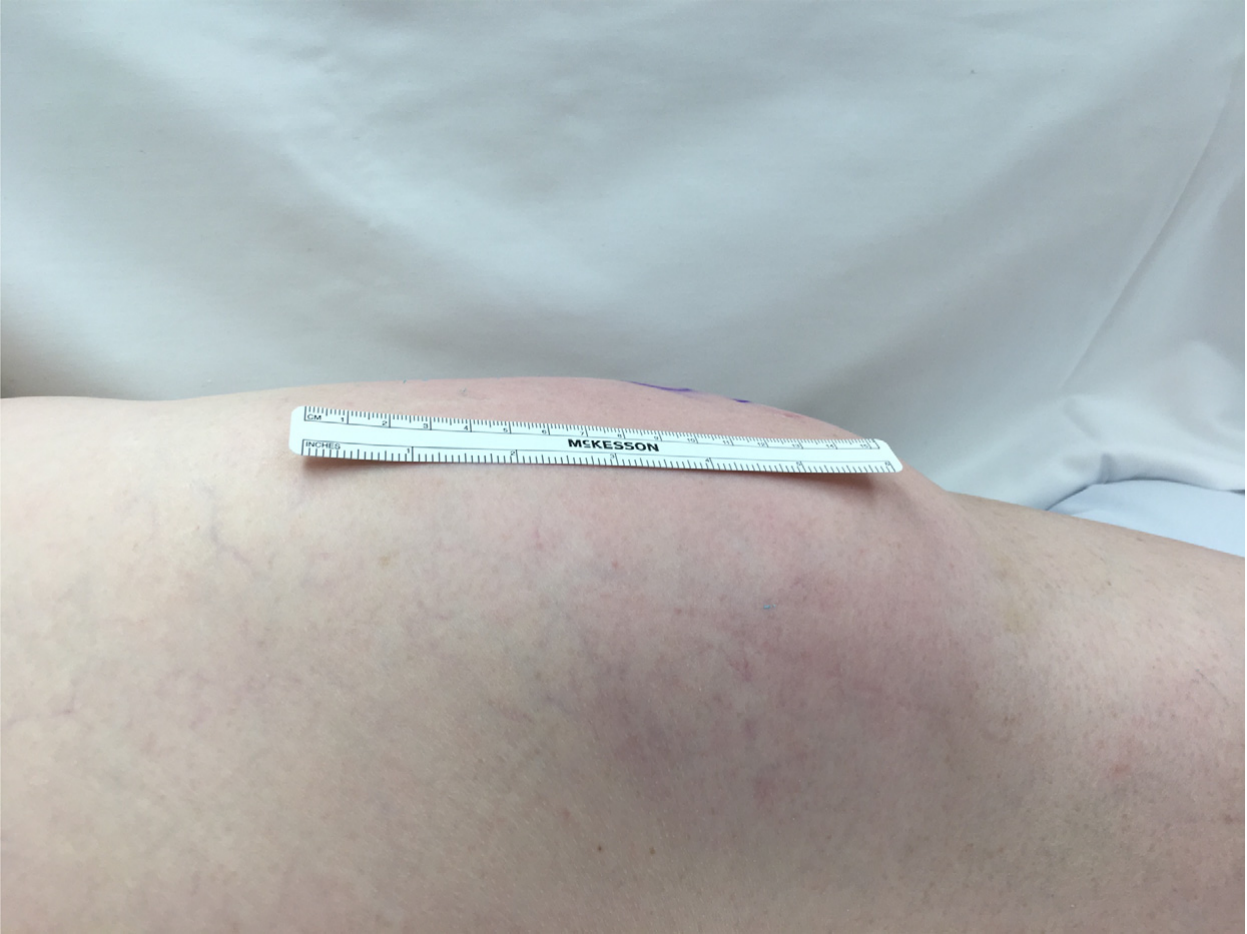
Intra‐op photograph.

**Figure 8:**
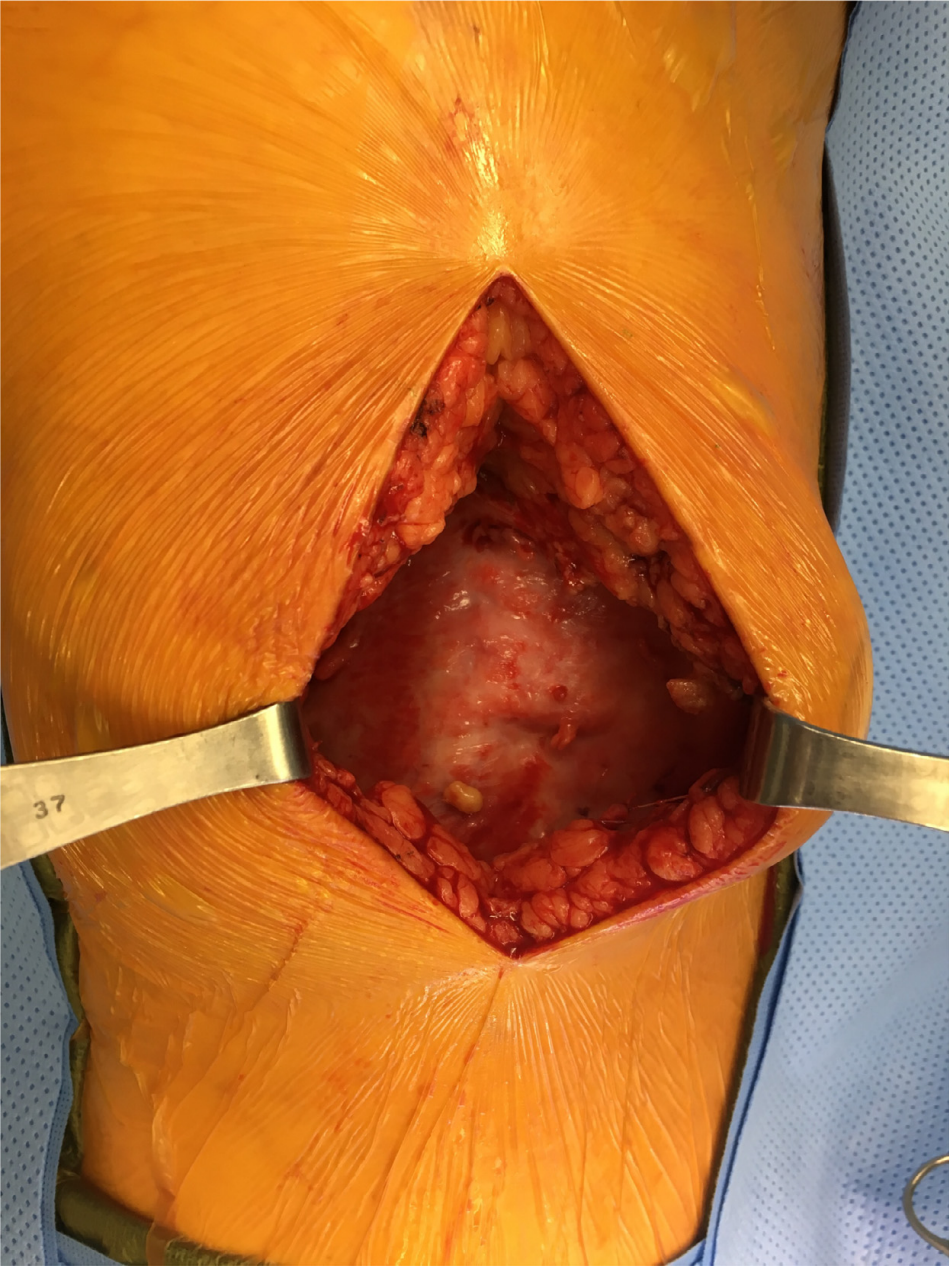
Intra‐op photograph.

Follow‐up with the trauma surgeon on postinjury day 17 included a focused review of systems and physical examination, and also a review of the MRI findings and a discussion of treatment options and their success rates. Based upon the Mellado and Bencardino classification pattern of lesions, the treatment options included ultrasound or computed tomography‐guided percutaneous drainage, sclerotherapy, and open debridement.^6^ Deciding upon the most appropriate treatment is often difficult.^8^ Once the lesion has been identified, treatment should consist of evacuation of the hematoma and necrotic material.^9^ Once a capsule has been identified, conservative or percutaneous treatment is unsuccessful^9^ (Figs. 6, 7, 8).

### Surgical technique

Diagnosis of MLLs is based on history and physical examination. These injuries often occur in association with fractures, such as of the pelvis and acetabulum, femur, and tibia fractures as a result of high‐energy mechanisms. MLLs may also occur in isolation, as in the illustrated case. The patient presents with a focal area of swelling and may or may not have associated pain or tenderness. A sensation of a fluid collection is often described as a “water balloon” under the skin.

On examination, the lesion often shows a focal area of swelling, but may be less visible in patients with a large body habitus. There may be associated ecchymosis and abrasions. Significant fluctuance is a hallmark, as the lesion is filled mainly by serous fluid. Because MLLs occur at the interface between subcutaneous fat and the deep fascia, the collection feels superficial. This is in contrast to a hematoma, which is typically deeper, less well defined, and less fluctuant. Even so, differentiating between MLL and a hematoma may not be possible by examination alone.

Further evaluation of the injury can be accomplished by MRI to help confirm the diagnosis, eliminate associated injuries, and also establish the size and location of the lesion for surgical planning.

Operative intervention is often recommended because spontaneous resolution is uncommon. The incision is planned in a location that will allow access to the entirety of the lesion. Multiple incisions may be necessary for a very large MLL. A large collection of serous to serosanguinous fluid is encountered after dissection through the subcutaneous fat. A pseudocapsule is present unless surgery is performed in the earliest stages. A very mature appearing capsule may be present in chronic lesions. The goal is to debride the surfaces of the interior of the lesion to produce bleeding and thereby a healing response to allow scarring of the surfaces to each other. Alternatively, it may be necessary to completely excise the pseudocapsule in the case of chronic lesions. Any devitalized tissue should be debrided as well. After thoroughly irrigating the wound, the cavity is then closed. The deep (fascial) layer is closed to the deep surface of the subcutaneous fat. It helps to imagine this as being analogous to sewing the inside surfaces of a balloon to each other in order to eliminate the cavity and allow the surfaces to heal to each other. This is performed over drains to prevent reaccumulation of fluid. As many drains as necessary should be placed to ensure that no isolated pockets can form which may allow recurrence of the MLL. The drains are brought out through the skin in a nondependent position away from the lesion, so that prolonged drainage through the skin punctures does not occur after pulling the drains. The drains are typically left until there is no further output.

### Outcome and follow‐up

**Figure 9:**
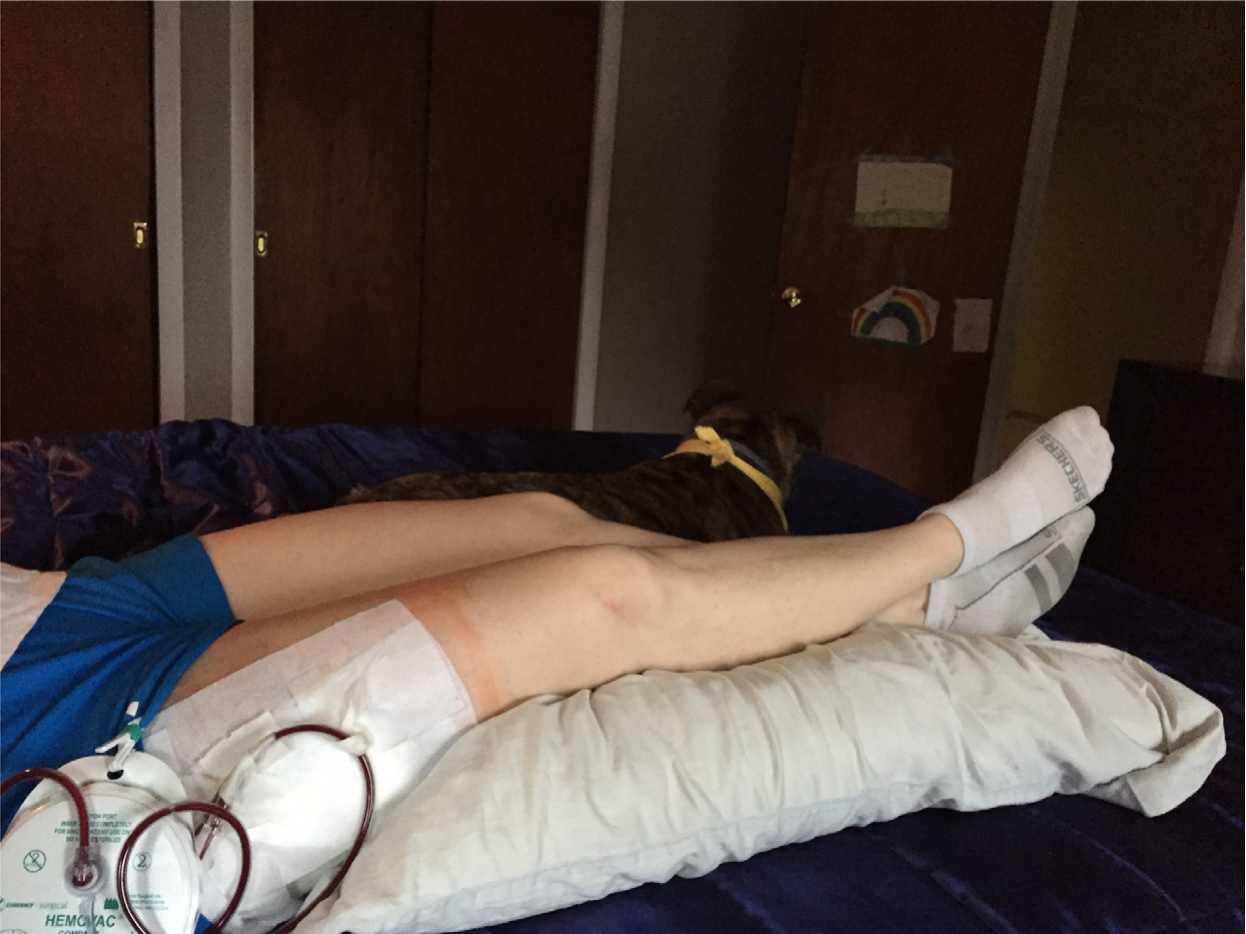
Postop day 0.

**Figure 10:**
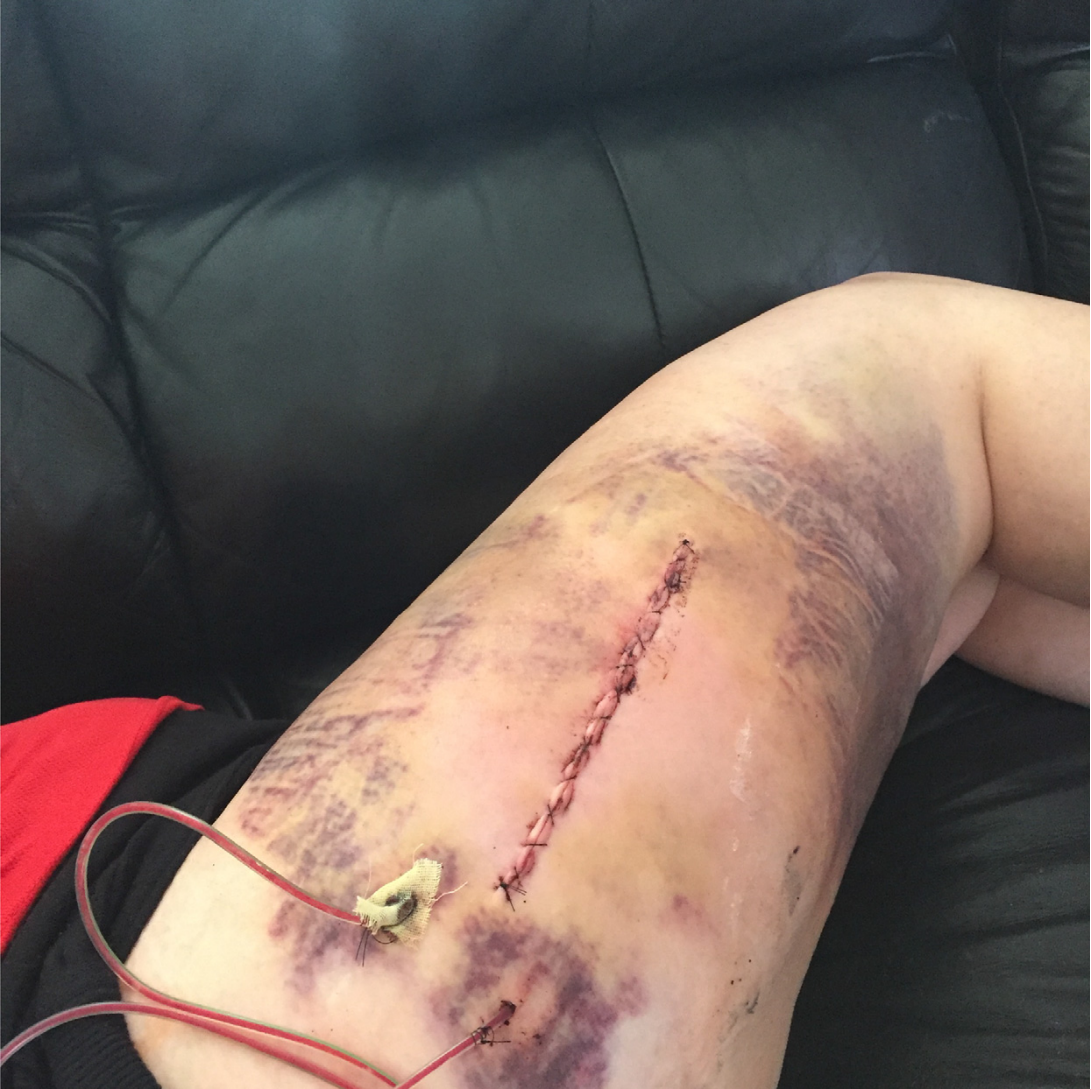
Postop day 3.

Postoperative activity is usually restricted until wounds are healed. Strenuous activity should be avoided to prevent shearing of the tissues and to allow healing of the cavity surfaces. Six weeks is likely sufficient, at which point activity can be progressed as tolerated. In this case, the postoperative course was uncomplicated (Fig. 9). The original dressing was removed on day 3 and replaced with gauze and paper tape over the drains and large plastic bandages over the remaining wound (Fig. 10).

**Figure 11:**
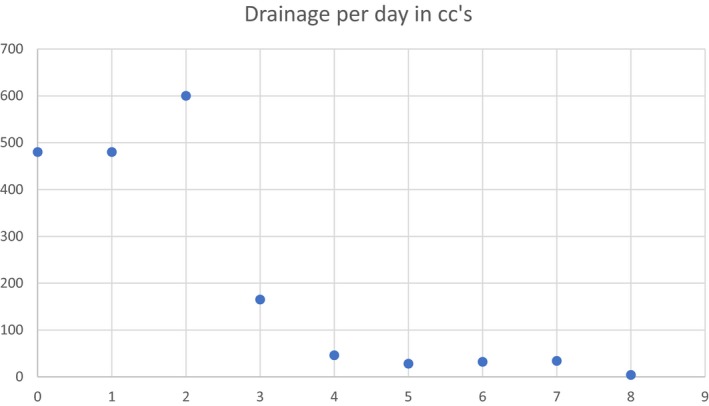
Hemovac drainage volume.

The output of the drains on the day of surgery was 480 cc, decreasing to minimal on the day they were removed. The total output from the drains was 1869 cc (see Fig. 11).

## Discussion

This case is clinically significant for three primary reasons. First, the Morel‐Lavallee lesions are rare, post‐traumatic, soft‐tissue degloving injuries commonly misdiagnosed as hematomas or ruptured bursa, and Morel‐Lavallee lesion is not widely known among professionals who work in the front line of emergency services and orthopedic surgeons.^4^, ^10^ If not treated in the acute and subacute setting, these lesions are often complicated by reaccumulation of fluid, infection, continued expansion, overlying tissue necrosis, chronic pain, and suboptimal patient outcomes.^9^, ^10^ The clinician needs to be aware of this injury, in order to provide appropriate patient care and treatment. It remains primarily a clinical diagnosis.^16^ This case was treated early, with excellent clinical outcomes.

Second, the initial treatment should include early drainage/debridement, which will most likely prevent recurrence and significantly shorten the clinical course.^10^ Open debridement has been shown to be a safe and effective method for treating Morel‐Lavallee lesions.^14^, ^17^

Third, the modality of choice for imaging is MRI for Morel‐Lavallee lesions.^8^, ^9^, ^16^ MRI offers multiplanar imaging and high contrast resolution to provide greater anatomic details.^6^ MRI can also assist in providing detailed information regarding the location within fascial planes and differentiating the six types of lesions.^14^ In this case, an MRI was obtained early and assisted the surgeon in making a decision to proceed with surgical intervention.

## Authorship

KM: contributed to the writing, editing, and revising of the manuscript. SD: contributed to the writing, editing, and revising of the manuscript.

## Conflict of Interest

None declared.
